# How is neighborhood social disorganization associated with diabetes outcomes? A multilevel investigation of glycemic control and self-reported use of acute or emergency health care services

**DOI:** 10.1186/s40842-018-0069-0

**Published:** 2018-10-19

**Authors:** Sarah D. Kowitt, Katrina E. Donahue, Edwin B. Fisher, Madeline Mitchell, Laura A. Young

**Affiliations:** 1 Peers for Progress and Department of Health Behavior, Gillings School of Global Public Health, University of North Carolina at Chapel Hill, Rosenau Hall, CB #7440, Chapel Hill, NC 27599-7440 USA; 2 Department of Family Medicine, University of North Carolina at Chapel Hill, Chapel Hill, NC 27599 USA; 3 Cecil G. Sheps Center for Health Services Research, University of North Carolina at Chapel Hill, Chapel Hill, NC 27599 USA; 4 Division of Endocrinology & Metabolism, School of Medicine, University of North Carolina at Chapel Hill, Chapel Hill, NC 27599 USA

**Keywords:** Health policy, Neighborhood, Environmental influences, Poverty, Emergency services, Social determinants, Psychosocial influences, Type 2 diabetes

## Abstract

**Background:**

Diabetes management is influenced by a number of factors beyond the individual-level. This study examined how neighborhood social disorganization (i.e., neighborhoods characterized by high economic disadvantage, residential instability, and ethnic heterogeneity), is associated with diabetes-related outcomes.

**Methods:**

We used a multilevel modeling approach to investigate the associations between census-tract neighborhood social disorganization, A1c, and self-reported use of acute or emergency health care services for a sample of 424 adults with type 2 diabetes.

**Results:**

Individuals living in neighborhoods with high social disorganization had higher A1c values than individuals living in neighborhoods with medium social disorganization (B = 0.39, *p* = 0.01). Individuals living in neighborhoods with high economic disadvantage had higher self-reported use of acute or emergency health care services than individuals living in neighborhoods with medium economic disadvantage (B = 0.60, *p* = 0.02).

**Conclusions:**

High neighborhood social disorganization was associated with higher A1c values and high neighborhood economic disadvantage was associated with greater self-reported use of acute or emergency health care services. Controlling for individual level variables diminished this effect for A1c, but not acute or emergency health care use. Comprehensive approaches to diabetes management should include attention to neighborhood context. Failure to do so may help explain the continuing disproportionate diabetes burden in many neighborhoods despite decades of attention to individual-level clinical care and education.

**Trial registration:**

For this study, we used baseline data from a larger study investigating the impacts on patient-centered outcomes of three different approaches to self-monitoring of blood glucose among 450 adults with non-insulin dependent type 2 diabetes living in North Carolina. This study was registered as a clinical trial on 1/7/2014 (https://clinicaltrials.gov/ct2/show/NCT02033499).

## Background

Diabetes is the seventh leading cause of death and associated with significant health complications [1]. Compared to individuals without diabetes, people with diabetes and particularly those with poor diabetes management, as evidenced through elevated glycemic control (A1c) are at greater risk for heart attack, stroke, kidney failure, and premature mortality [1]. Moreover, an estimated one in seven health care dollars is attributed to diabetes—resulting in a $327 billion total estimated cost [2]. For these reasons, diabetes management poses a significant problem that needs to be addressed, particularly for individuals in the Southeastern U.S. who experience greater rates of diabetes than the general population (colloquially called the “diabetes belt”) [3].

Moving beyond the “self” in “diabetes self-management,” research has shown that diabetes management is influenced by a number of ecological factors beyond the individual and that interventions focused solely on the individual are often insufficient to improve glycemic control over the long-term [4]. There is growing empirical evidence that even after controlling for individual level socioeconomic status (SES) and race/ethnicity, aspects of the neighborhood are associated with health status [5] and glycemic control [6] among individuals with diabetes, as well as risk of developing diabetes [7–10]. While most of the evidence to date has been observational, in one of the only randomized studies of neighborhood poverty, researchers found that individuals randomized to live in low poverty neighborhoods were less likely to develop diabetes than individuals randomized to live in high poverty neighborhoods [8]. In addition to the growing body of empirical evidence, researchers have also conceptualized *how* neighborhood characteristics are associated with health outcomes.

Social disorganization theory suggests that in addition to neighborhood disadvantage—a well-documented predictor of poor health status [11]—other features of neighborhoods, such as residential instability and ethnic heterogeneity (i.e., an index of neighborhood diversity) influence health outcomes [12]. Specifically, this theory posits that neighborhoods with social disorganization (i.e., neighborhoods characterized by high economic disadvantage, residential instability, and ethnic heterogeneity) have lower social control and collective efficacy and higher violence and crime. While this theory has mostly been applied to violence or substance use [12], researchers have hypothesized that neighborhood social disorganization (NSD) may also affect physical health outcomes [13] through its influence on: 1) health-related behaviors by constraining diffusion of health information and reducing social control over deviant health-related behavior; 2) access to services and amenities by affecting residents’ ability to lobby for provision of services and use of such services (e.g., health care services) that are directly related to health; and 3) psychosocial processes by influencing levels of affective support, stress, self-esteem and mutual respect—all of which are associated with immune response and overall health [14]. In a study of cardiometabolic risk factors among African Americans, for example, a composite measure of NSD was associated with presence of metabolic syndrome in women (defined as having 3 of 5 of the following risk factors: elevated serum triglycerides, fasting plasma glucose, blood pressure, waist circumference, and decreased high density lipoprotein cholesterol), even after adjusting for age, health behaviors, income, education, and family size [15].

While researchers have often examined economic disadvantage and residential instability as metrics of neighborhood functioning, the concept of ethnic heterogeneity is specific to social disorganization theory. Ethnic heterogeneity is hypothesized to affect health outcomes because it can contribute to lack of communication between neighborhoods, hinder social ties, and increase social isolation, leading to less social control and reduced neighborhood collective efficacy [12]. Indeed, a number of researchers have found increasing ethnic heterogeneity to be associated with increased rates of dating violence victimization [16], increased rates of assault, juvenile violence, or violent crime [17–20], and weakened perceptions of collective efficacy [21]. Even in the study described above examining cardiometabolic risk factors among African Americans, the composite measure of NSD included dimensions assessing ethnic heterogeneity [15]. However, other research suggests that associations between increasing ethnic heterogeneity and worse health outcomes may be misleading due to methodological artifacts or other confounding variables [22, 23]. Many arguments can also be made for the benefits of neighborhood racial and ethnic diversity, such as increased cultural sensitivity among residents, decreased racial and ethnic prejudice, broadened social networks, and increased social growth [24]. Given that social disorganization theory was first developed in 1969, it is possible that ethnic heterogeneity may no longer be relevant for health or social processes and further research is needed.

Despite the growing body of evidence examining associations between neighborhood characteristics and health outcomes among individuals with diabetes, few studies have used behavioral health theories to examine associations, which can help guide selection of appropriate variables for analyses and can also facilitate comparisons of results across studies. Moreover, no studies to our knowledge have examined NSD and its association with diabetes-related outcomes. Therefore, in the current study, we use social disorganization theory to examine how NSD may be associated with two diabetes outcomes: A1c and self-reported use of emergency and acute health care services. We chose A1c as an outcome given its importance as a marker of glycemic control and diabetes management [1]. In addition, we chose use of acute or emergency health care services given its impact on health care expenditures [2] and because few studies have examined how neighborhood characteristics may be associated with use of acute or emergency health care services among individuals with type 2 diabetes. Based on prior research [5–10, 25], we hypothesized that NSD would be associated with both A1c and self-reported use of emergency and acute health care services.

## Methods

### Data source

For this study, we used baseline data from a larger parent study investigating the impacts on patient-centered outcomes of three different approaches to self-monitoring of blood glucose among 450 adults with non-insulin dependent type 2 diabetes living in North Carolina [26, 27]. Patients from primary care practices within the central North Carolina area were recruited to take part in the parent study. Participants were aged 30 or over with an A1c between 6.5 and 9.5% within the 6 months preceding screening. All measurements were collected as part of the larger parent study. For the present study, we used baseline data from all participants. Baseline data were collected betwen January 2014 and June 2014. The University of North Carolina at Chapel Hill Institutional Review Board approved the parent study, as well as the present study.

### Measures

#### A1c

To assess A1c, we collected blood at the time of the patient’s baseline visit and measured total glycated hemoglobin using a published formula by the processing laboratory.

#### Self-reported use of acute or emergency health care services

To assess use of acute or emergency health care services, we asked participants “in the last year, how many times have you” 1) “gone to an urgent care clinic?” 2) “been seen in the Emergency Room?” 3) “been hospitalized overnight?” and 4) “had someone call EMS for you?”. We summed responses to create a count of how many visits a participant made to the emergency room, urgent care, hospital, or ambulatory care in the previous year.

#### NSD

Of the 450 participants, we were able to geocode 436 addresses into 200 census tracts; the remaining 14 addresses were PO boxes, which we excluded for this study. We then downloaded 2013 American Community Survey census tract data and merged these with the individual-level dataset. Based on previous research [13, 28], we used seven census indicators to represent the three components of NSD. These census indicators were used to assess *neighborhood economic disadvantage* (i.e., the proportion of female-headed families, the proportion of individuals in poverty, the proportion of households receiving public assistance, and the proportion of unemployed individuals) [13, 28], *neighborhood residential instability *(i.e., the proportion of renter-occupied homes vs. owner-occupied and the proportion of residents who had lived in the neighborhood for less than 5 years) [28], and *neighborhood ethnic heterogeneity* (i.e., calculated as the sum of the squared proportions of each racial / ethnic group in the neighborhood subtracted from one) [29].

We analyzed NSD in two ways. First, in line with previous research [15], we averaged these seven indicators to create a score that ranged from 0 to 1 with higher values indicating greater NSD (Cronbach’s alpha = 0.76). We then created tertiles of NSD with neighborhoods with low NSD defined as one standard deviation below the mean and neighborhoods and high NSD defined as one standard deviation above the mean—as has been done in previous research [28, 30–32]. Second, we examined these seven indicators separately as variables representing neighborhood economic disadvantage, neighborhood residential instability, and neighborhood ethnic heterogeneity, as has also been done previously [28]. Similar to the first approach, we averaged respective indicators and created tertiles with low defined as one standard deviation below the mean and high defined as one standard deviation above the mean.

#### Psychosocial and clinical variables

Psychosocial and clinical variables included: years with diabetes, diabetes distress, diabetes empowerment, self-care, and number of comorbidities. We measured *years with diabetes* by asking participants how long ago they were diagnosed with diabetes (in years). We measured *diabetes distress* with 20 items form the Problem Areas in Diabetes (PAID) scale, which assesses diabetes-specific emotional distress, including guilt, anger, depressed mood, worry, and fear [33]. Each item has five possible answers ranging from 0 (representing “no problem”) to 4 (“a serious problem”) [33]. We added the scores and multiplied by 1.25 to generate a total score between 0 and 100, with higher values indicating more distress [33]. We measured *diabetes empowerment* with eight items from Diabetes Empowerment Scale-Short Form (DES-SF), which assesses one’s confidence in managing, coping, and making positive choices about diabetes care [34]. Each item has 5 response options (1 = strongly disagree to 5 = strongly agree), which we averaged for a scale ranging from 1 to 5, with higher values indicating higher diabetes empowerment [34]. We measured *self-care* with items from the Summary of Diabetes Self-Care Activities, which is a brief self-report questionnaire of general diet, specific diet, exercise, blood-glucose testing, foot care, and smoking [35]. For the purposes of this study, we only included self-care items for general diet, specific diet, exercise, blood-glucose testing, and footcare [35]. For each item, response options range from 0 to 7, indicating the frequency with which activities had been performed over the previous week (e.g., participating in at least 30 min of physical activities). We created a total mean score by averaging all items [35]. We measured *comorbidities* by asking participants to self-report other conditions, including chronic back pain, heart disease, high blood pressure, lung disease, stroke, high cholesterol, kidney disease, liver disease, anemia or other blood disease, cancer, depression / anxiety, arthritis, autoimmune disease, and stomach or bowel disease. We created a total score by summing the number of comorbidities; higher values indicate more comorbidities.

#### Demographic variables

Demographic variables included education (categorized as completed some high school, high school graduate, some college, college degree, or graduate degree), age, sex, ethnicity (Latino vs. not Latino), and race (options for American Indian or Alaska Native, Asian, Black or African American, Native Hawaiian or Pacific Islander, White, Other, and Mixed). Given the limited number of participants not identifying as Black or White, we collapsed race into the following categories: Black, White, and Other.

### Data analysis

We collected data from January 2014 to June 2014 and conducted data analysis between September 2015 and February 2016. We used SAS version 9.3 survey procedures (SAS Inc., Cary, NC, USA) for descriptive statistics and multilevel modeling (Proc Mixed and Proc Glimmix). Of the 450 participants with baseline data, we dropped data for 14 participants (3.1%) who could not be geocoded and 12 participants (3%) who were missing data on any of the other variables examined, resulting in an analytic sample of 424 participants. We set critical α = .05 and used 2-tailed statistical tests.

#### Multilevel models

We applied two-level random intercept models to assess the associations between neighborhood characteristics and individual-level diabetes management outcomes [36].

Before constructing the multilevel models, we examined descriptive statistics and unadjusted associations between NSD and each outcome. Then we constructed the multilevel models in steps of increasing complexity. First, we constructed a null model to quantify the between and within-tract variance of the outcomes, or in other words to estimate the intraclass correlation (ICC). This model was not presented in the tables. Next, we constructed a multilevel random intercept model (Model 1), with individual-level demographic predictors modeled as fixed effects, to examine the influence of individual-level characteristics on our outcomes. Third, we entered individual level demographic, psychosocial, and clinical variables into the model (Model 2) as fixed effects to determine the influence of psychosocial and clinical factors on our outcomes. Finally, we added neighborhood-level contextual factors in two ways: NSD modeld as three separate variables (Model 3) and NSD modeled as a composite variable (Model4). To model A1c, we used a linear multilevel modeling approach. For use of acute or emergency health care services (i.e., counts data), we used a series of Poisson models to account for the non-normal distribution of the data [37].

Finally, to determine if the individual-level variables mediated or confounded the relationship between NSD and our outcomes, we examined whether there was a significant relationship between NSD and our outcomes with and without individual-level variables in the model. We also ran sensitivity analyses to see if effects varied when individual-level factors were entered into the model as random effects (rather than fixed effects) and used Akaike Information Criteria and Bayesian Information Criterion values to determine which model had the best fit (smaller values indicate better fit).

In the results, regression coefficients (“B”) and 95% confidence intervals (CIs) are presented. Within each model, the referent group was chosen as the group with the largest number of participants.

## Results

### Descriptive statistics

On average participants were 60.5 years old and majority White (63.9%) (Table 1). Most participants had completed some college (36.3%), had a college degree (20.8%), or had a graduate degree (13.4%). Mean A1c was 7.5 and, on average, participants reported using 1 acute or emergency health care service in the previous year (mean: 1.2, SD: 2.3). Participants also reported having between 3 and 4 comorbidities, on average (mean: 3.4, SD: 1.9). While most participants lived in neighborhoods with medium NSD (70.8%), an appreciable minority lived in neighborhoods with high NSD (12.5%), defined as one standard deviation above the mean.

**Table 1:** Participant characteristics, *n* = 424

Characteristic	N (%) or mean (SD)
Age, mean (SD)	60.5 (11.5)
Sex	
Female	224 (52.8)
Male	200 (47.2)
Educational level	
Some HS	24 (5.7)
HS grad or GED	101 (23.9)
Some college	154 (36.3)
College degree	88 (20.8)
Grad degree	57 (13.4)
Race	
White	271 (63.9)
Black	138 (32.6)
Other	15 (3.5)
Latino	
No	417 (98.4)
Yes	7 (1.7)
Duration of diabetes, mean (SD)	8.2 (7.6)
Diabetes empowerment, mean (SD)	4.3 (0.5)
Diabetes self-care, mean (SD)	3.4 (1.4)
Diabetes distress, mean (SD)	10.5 (12.8)
Comorbidities, mean (SD)	3.4 (1.9)
Self-reported use of acute or emergency health care services, mean (SD)	1.2 (2.3)
A1c, mean (SD)	7.5 (1.1)
Neighborhood economic disadvantage^a^	
Low	52 (12.3)
Medium	314 (74.1)
High	58 (13.7)
Neighborhood residential instability^a^	
Low	61 (14.4)
Medium	288 (67.9)
High	75 (17.7)
Neighborhood ethnic heterogeneity^a^	
Low	79 (18.6)
Medium	265 (62.5)
High	80 (18.9)
NSD^a^	
Low	71 (16.8)
Medium	300 (70.8)
High	53 (12.5)

NSD refers to neighborhood social disorganization

^a^ For all of the neighborhood variables, low was defined as one standard deviation below the mean, and high was defined as one standard deviation above the mean

### Simple, unadjusted effects of neighborhood disadvantage

#### A1c

In an unadjusted model (Table 2), there were no associations between A1c and neighborhood economic disadvantage, neighborhood residential instability, or neighborhood ethnic heterogeneity, that is, the individual scales that comprised NSD. However, turning to the composite measure of NSD, individuals living in neighborhoods with high NSD had higher A1c values than individuals living in neighborhoods with medium NSD (B = 0.47, *p* = 0.003), whereas individuals living in neighborhoods with low NSD had similar A1c values compared to individuals living in neighborhoods with medium NSD (B = 0.17, *p* = 0.21).

**Table 2:** Unadjusted effects of neighborhood variables on outcomes, *n* = 424

Variable	A1c^a^		Self-reported use of acute or emergency health care services^a^	
	Regression Coefficient B (95% CI)	*p*-value	Regression Coefficient B (95% CI)	*p*-value
Neighborhood economic disadvantage				
Low	0.26 (−0.06, 0.58)	0.11	−0.55 (−1.09, − 0.01)	0.05
Medium	REF		REF	
High	0.23 (−0.08, 0.54)	0.15	0.49* (0.03, 0.95)	0.04
Neighborhood residential instability				
Low	0.07 (− 0.23, 0.38)	0.63	− 0.42 (− 0.94, 0.09)	0.11
Medium	REF		REF	
High	0.19 (− 0.09, 0.47)	0.18	−0.22 (− 0.69, 0.24)	0.33
Neighborhood ethnic heterogeneity				
Low	−0.002 (− 0.28, 0.27)	0.98	− 0.27 (− 0.74, 0.20)	0.25
Medium	REF		REF	
High	0.16 (−0.12, 0.43)	0.26	0.02 (−0.43, 0.46)	0.94
NSD (composite measure)				
Low	0.17 (−0.10, 0.45)	0.21	−0.25 (− 0.72, 0.22)	0.29
Medium	REF		REF	
High	0.47** (0.16, 0.78)	0.003	0.04 (−0.44, 0.53)	0.86

NSD refers to neighborhood social disorganization

* *p* < 0.05

** *p* < 0.01

^a^Model adjusts for clustering of observations within census tract, but does not adjust for any individual-level demographic, psychosocial, or clinical variables. Each neighborhood variable was analyzed separately

#### Use of acute or emergency health care services

Turning to self-reported use of acute or emergency health care services (Table 2), individuals living in neighborhoods with high neighborhood economic disadvantage reported greater use of acute or emergency health care services (B = 0.49, *p* = 0.04) than individuals living in neighborhoods with medium economic disadvantage, while the individual measures of neighborhood residential instability and neighborhood ethnic heterogeneity were not significantly related to utilization. There were no associations with the composite measure of NSD.

### Multilevel models examining A1c

Table 3 provides information from the four different models regarding individual and neighborhood-level predictors of A1c. In the null model, 3.3% of the total variability in A1c was due to variation between neighborhoods, while the remainder of the variation in A1c was due to variation within neighborhoods, that is, individual variation.

**Table 3:** Effects of neighborhood variables and correlates on A1c, *n* = 424

Variable^a^	Model 1		Model 2		Model 3		Model 4	
	Regression Coefficient	*p*-value	Regression Coefficient	*p*-value	Regression Coefficient	*p*-value	Regression Coefficient	*p*-value
	B (95% CI)		B (95% CI)		B (95% CI)		B (95% CI)	
Intercept	7.55 (7.48, 7.66)	< 0.001	7.55 (7.45, 7.65)	< 0.001	7.55 (7.45, 7.65)	< 0.001	7.55 (7.45, 7.64)	< 0.001
Sex								
Female	REF		REF		REF		REF	
Male	0.01 (−0.20, 0.22)	0.91	0.02 (− 0.18, 0.22)	0.84	0.02 (− 0.19, 0.22)	0.87	0.01 (− 0.19, 0.22)	0.90
Age	−0.01 (− 0.01, 0.00)	0.28	0.00 (− 0.01, 0.01)	0.53	0.00 (− 0.01, 0.01)	0.48	0.00 (− 0.01, 0.01)	0.48
Race								
White	REF		REF		REF		REF	
Black	0.02 (−0.21, 0.25)	0.86	−0.03 (− 0.26, 0.19)	0.77	−0.06 (− 0.30, 0.18)	0.61	−0.07 (− 0.30, 0.16)	0.54
Other	0.39 (−0.17, 0.96)	0.17	0.34 (−0.2, 0.88)	0.22	0.26 (− 0.29, 0.81)	0.36	0.25 (− 0.30, 0.79)	0.37
Educational level								
Some HS	0.16 (− 0.31, 0.62)	0.51	0.03 (−0.42, 0.48)	0.91	0.05 (−0.41, 0.51)	0.83	0.05 (− 0.40, 0.50)	0.84
HS grad	−0.05 (− 0.32, 0.22)	0.73	− 0.01 (− 0.27, 0.25)	0.96	−0.01 (− 0.28, 0.25)	0.93	− 0.01 (− 0.26, 0.25)	0.97
Some college	REF		REF		REF		REF	
College grad	0.14 (− 0.14, 0.42)	0.34	0.10 (− 0.17, 0.37)	0.45	0.09 (−0.18, 0.37)	0.49	0.13 (−0.14, 0.4)	0.34
Grad	−0.02 (− 0.35, 0.31)	0.90	− 0.05 (− 0.36, 0.27)	0.77	− 0.06 (− 0.38, 0.26)	0.71	− 0.03 (− 0.34, 0.29)	0.87
Years with diabetes	–	–	0.04^***^ (0.02, 0.05)	< 0.001	0.04^***^ (0.02, 0.05)	< 0.001	0.04^***^ (0.02, 0.05)	< 0.001
Diabetes distress	–	–	0.02^***^ (0.01, 0.03)	< 0.001	0.02^***^ (0.01, 0.03)	< 0.001	0.02^***^ (0.01, 0.03)	< 0.001
Diabetes empowerment	–	–	0.24^*^ (0.03, 0.45)	0.03	0.25^*^ (0.04, 0.46)	0.02	0.24^*^ (0.03, 0.45)	0.03
Self-reported use of acute or emergency health care services	–	–	− 0.01 (− 0.06, 0.03)	0.61	− 0.01 (− 0.06, 0.04)	0.65	− 0.01 (− 0.06, 0.03)	0.60
Self-care	–	–	− 0.10^*^ (− 0.18, − 0.02)	0.02	−0.10^*^ (− 0.18, − 0.02)	0.02	−0.10^*^ (− 0.18, − 0.02)	0.01
Comorbidities	–	–	− 0.07^*^ (− 0.13, − 0.01)	0.03	− 0.06^*^ (− 0.12, 0.00)	0.04	−0.06^*^ (− 0.12, 0.00)	0.04
Neighborhood economic disadvantage								
Low	–	–	–	–	0.20 (− 0.14, 0.54)	0.25	–	–
Medium	–	–	–	–	REF		–	–
High	–	–	–	–	0.11 (− 0.21, 0.42)	0.51	–	–
Neighborhood residential instability								
Low	–	–	–	–	0.01 (− 0.31, 0.33)	0.95	–	–
Medium	–	–	–	–	REF		–	–
High	–	–	–	–	0.18 (− 0.10, 0.46)	0.21	–	–
Neighborhood ethnic heterogeneity								
Low	–	–	–	–	− 0.03 (− 0.3, 0.25)	0.84	–	–
Medium	–	–	–	–	REF		–	–
High	–	–	–	–	0.01 (− 0.26, 0.28)	0.94	–	–
NSD (composite measure)								
Low	–	–	–	–	–	–	0.09 (− 0.18, 0.36)	0.52
Medium	–	–	–	–	–	–	REF	
High	–	–	–	–	–	–	0.39^*^ (0.08, 0.69)	0.01

Model 1 - Individual demographic variables. Model 2 - Individual demographic, psychosocial, and clinical variables. Model 3 - Individual demographic, psychosocial, and clinical variables and separate NSD measures. Model 4 - Individual demographic, psychosocial, and clinical variables and composite NSD measure

NSD refers to neighborhood social disorganization

* *p* < 0.05

*** *p* < 0.001

^a^In all models, variables were grand mean centered to increase interpretability

#### Model 1

We observed no significant relationships between A1c and demographic characteristics (i.e., race, age sex, educational level).

#### Model 2

Several psychosocial and clinical variables, including greater years with diabetes (B = 0.04, *p* < 0.001), greater diabetes distress (B = 0.02, *p* < 0.001), and greater diabetes empowerment (B = 0.24, *p* = 0.03) were significantly associated with higher A1c values, while greater self-care (B = − 0.10, *p* = 0.02) and comorbidities (B = − 0.07, *p* = 0.03) were associated with lower A1c values.

#### Model 3

There were no associations between A1c and neighborhood economic disadvantage, neighborhood residential instability, or neighborhood ethnic heterogeneity, that is, the individual scales that comprised NSD. Greater years with diabetes (B = 0.04, *p* < 0.001), greater diabetes distress (B = 0.02, *p* < 0.001), and greater diabetes empowerment (B = 0.25, *p* = 0.02) were all still associated with higher A1c values, while greater self-care (B = − 0.10, *p* = 0.02) and greater comorbidities (B = − 0.06, *p* = 0.04) were still associated with lower A1c values.

#### Model 4

As hypothesized, individuals who lived in neighborhoods with high NSD (the composite measure) had higher A1c values (B = 0.39, *p* = 0.01), when compared to individuals who lived in neighborhoods with medium NSD. There was no difference in A1c values, however, for individuals living in neighborhoods with low NSD, compared to individuals living in neighborhoods with medium NSD (B = 0.09, *p* = 0.52). Greater years with diabetes (B = 0.04, *p* < 0.001), greater diabetes distress (B = 0.02, *p* = 0.001), and greater diabetes empowerment (B = 0.24, *p* = 0.03) were all still associated with higher A1c values, while greater self-care (B = − 0.10, *p* = 0.01) and greater comorbidities (B = − 0.06, *p* = 0.04) were still associated with lower A1c values.

#### Comparisons to unadjusted effects

The role of the composite measure of NSD was reduced somewhat by the inclusion of other variables in the models. Compared to the unadjusted effects in Table 2, the regression coefficient comparing high and medium NSD declined in magnitude (from B = 0.47 to B = 0.39) and level of significance (from *p* = 0.003 to p = 0.01).

### Multilevel models examining self-reported use of acute or emergency health care services

For acute or emergency use of health care services, we used a series of Poisson models to model the non-normal data. In Poisson regression, there is no estimate of ICC [37]. As a result, we compared the magnitude of clustering by comparing an empty fixed model with a fixed intercept and no clustering of the data to a random intercept null model with clustering specified [37]. Results indicated that the model that incorporated clustering had lower Akaike Information Criteria and Bayesian Information Criterion values, suggesting better fit. Table 4 provides information from the four different models regarding individual and neighborhood-level predictors of acute or emergency health care utilization.

**Table 4:** Effects of neighborhood variables and correlates on self-reported use of acute or emergency health care services, *n* = 424

Variable^a^	Model 1		Model 2		Model 3		Model 4	
	Regression Coefficient	*p*-value	Regression Coefficient	*p*-value	Regression Coefficient	*p*-value	Regression Coefficient	*p*-value
	B (95% CI)		B (95% CI)		B (95% CI)		B (95% CI)	
Intercept	−0.31 (− 0.53, − 0.10)	0.003	− 0.34 (− 0.53, − 0.14)	0.001	−0.33 (− 0.53, − 0.14)	0.001	−0.34 (− 0.54, − 0.14)	0.001
Sex								
Female	REF		REF		REF		REF	
Male	−0.07 (−0.15, 0.15)	0.52	0.12 (−0.11, 0.35)	0.30	0.12 (−0.11, 0.35)	0.32	0.12 (−0.11, 0.35)	0.32
Age	−0.03^***^ (− 0.02, − 0.02)	< 0.001	−0.04^***^ (− 0.05, − 0.02)	< 0.001	−0.03^***^ (− 0.05, − 0.02)	< 0.001	−0.04^***^ (− 0.05, − 0.02)	< 0.001
Race								
White	REF		REF		REF		REF	
Black	0.01 (−0.27, 0.26)	0.91	0.1 (−0.16, 0.37)	0.45	0.05 (−0.22, 0.32)	0.71	0.1 (−0.17, 0.36)	0.48
Other	−0.21 (− 0.31, 0.44)	0.52	− 0.26 (− 0.94, 0.41)	0.44	−0.31 (− 1.00, 0.37)	0.37	−0.27 (− 0.95, 0.4)	0.43
Educational level								
Some HS	0.03 (−0.41, 0.53)	0.91	−0.13 (− 0.65, 0.39)	0.63	− 0.19 (− 0.71, 0.33)	0.47	−0.13 (− 0.65, 0.39)	0.63
HS grad	0.33^*^ (− 0.27, 0.59)	0.02	0.16 (−0.12, 0.44)	0.27	0.13 (−0.16, 0.41)	0.38	0.16 (−0.12, 0.44)	0.27
Some college	REF		REF		REF		REF	
College grad	−0.20 (−0.13, 0.12)	0.22	−0.24 (− 0.58, 0.09)	0.16	− 0.25 (− 0.59, 0.08)	0.14	−0.24 (− 0.58, 0.1)	0.16
Grad	−0.04 (− 0.34, 0.34)	0.84	−0.15 (− 0.53, 0.23)	0.43	−0.12 (− 0.50, 0.26)	0.52	−0.15 (− 0.53, 0.23)	0.43
Years with diabetes	–	–	0.00 (− 0.02, 0.02)	0.91	0.00 (− 0.02, 0.02)	0.92	0.00 (− 0.02, 0.02)	0.91
Diabetes distress	–	–	0.00 (− 0.01, 0.01)	0.33	0.01 (0.00, 0.02)	0.26	0.00 (−0.01, 0.01)	0.34
Diabetes empowerment	–	–	0.06 (−0.18, 0.3)	0.60	0.07 (−0.17, 0.31)	0.58	0.06 (−0.18, 0.3)	0.61
A1c	–	–	−0.05 (− 0.17, 0.07)	0.42	−0.05 (− 0.17, 0.07)	0.38	−0.05 (− 0.17, 0.07)	0.41
Self-care	–	–	−0.06 (− 0.15, 0.04)	0.23	−0.05 (− 0.15, 0.04)	0.25	−0.06 (− 0.15, 0.03)	0.21
Comorbidities	–	–	0.23^***^ (0.17, 0.3)	< 0.001	0.23^***^ (0.17, 0.29)	< 0.001	0.23^***^ (0.17, 0.3)	< 0.001
Neighborhood economic disadvantage								
Low	–	–	–	–	−0.34 (− 0.92, 0.25)	0.26	–	–
Medium	–	–	–	–	REF		–	–
High	–	–	–	–	0.60^*^ (0.10, 1.09)	0.02	–	–
Neighborhood residential instability								
Low	–	–	–	–	0.01 (− 0.55, 0.57)	0.97	–	–
Medium	–	–	–	–	REF		–	–
High	–	–	–	–	− 0.39 (− 0.87, 0.09)	0.11	–	–
Neighborhood ethnic heterogeneity								
Low	–	–	–	–	−0.08 (− 0.55, 0.39)	0.74	–	–
Medium	–	–	–	–	REF		–	–
High	–	–	–	–	− 0.08 (− 0.51, 0.36)	0.73	–	–
NSD (composite measure)								
Low	–	–	–	–	–	–	− 0.04 (− 0.50, 0.42)	0.87
Medium	–	–	–	–	–	–	REF	
High	–	–	–	–	–	–	0.08 (− 0.40, 0.56)	0.75

Model 1 - Individual demographic variables. Model 2 - Individual demographic, psychosocial, and clinical variables. Model 3 - Individual demographic, psychosocial, and clinical variables and separate NSD measures. Model 4 - Individual demographic, psychosocial, and clinical variables and composite NSD measure

NSD refers to neighborhood social disorganization

* *p* < 0.05

** *p* < 0.01

*** *p* < 0.001

^a^In all models, variables were grand mean centered to increase interpretability

#### Model 1

We observed no significant relationships between self-reported use of acute or emergency health care services and some demographic characteristics (i.e., race, age sex, educational level), however, increasing age was negatively associated with self-reported use of acute or emergency health care service (B = − 0.03, *p* < 0.001). In addition, individuals with a high school degree reported greater use of acute or emergency health care services (B = 0.33, *p* = 0.02), compared to individuals with some college.

#### Model 2

Turning to psychosocial and clinical variables, greater comorbidities (B = 0.23, *p* < 0.001) was associated with greater self-reported use of acute or emergency health care services. Age was still negatively associated with self-reported use of acute or emergency health care services (B = − 0.04, *p* < 0.001).

#### Model 3

In the disaggregated evaluation of NSD indicators (Model 3), individuals who lived in neighborhoods with high economic disadvantage reported using acute or emergency health care services more than individuals who lived in neighborhoods with medium economic disadvantage (B = 0.60, *p* = 0.02). Age was still negatively associated with self-reported use of acute or emergency health care services (B = − 0.03, *p* < 0.001). In addition, greater comorbidities was still associated with greater self-reported use of acute or emergency health care services (B = 0.23, *p* < 0.001).

#### Model 4

Turning finally to the composite measure of NSD, it showed no association with self-reported use of acute or emergency health. Age was still negatively associated with self-reported use of acute or emergency health care services (B = − 0.04, *p* < 0.001). In addition, greater comorbidities was still associated with greater self-reported use of acute or emergency health care services (B = 0.23, *p* < 0.001).

#### Comparisons to unadjusted effects

The role of neighborhood economic disadvantage in explaining acute/emergency utilization was not significantly altered by the inclusion of other variables in the models. Compared to the unadjusted model in Table 2, the regression coefficient comparing high and medium neighborhood economic disadvantage was of similar magnitude (from B = 0.49 to B = 0.60) and level of significance (from *p* = 0.04 to *p* = 0.02).

### Model diagnostics

As a sensitivity analysis, we also entered in select individual-level variables as random effects into the models. However, when entered, the respective models for each outcome failed to converge, thereby indicating that this may not be an appropriate way to model the data. Additionally, we compared the Akaike Information Criteria and Bayesian Information Criterion values from the different models for A1c and use of acute or emergency health care services. These indicators suggested that the models with the composite and individual NSD variables respectively (Model 4 for A1c and Model 3 for acute or emergency health care service use) demonstrated the best fit (smallest Akaike Information Criteria and Bayesian Information Criterion values).

## Discussion

In this study among individuals with type 2 diabetes, we found individuals living in neighborhoods with high NSD (a composite of economic, residential, and racial / ethnic diversity indicators) had greater A1c values than individuals living in neighborhoods with medium NSD and that individuals living in neighborhoods with high economic disadvantage had higher self-reported use of acute or emergency health care services than individuals living in neighborhoods with medium economic disadvantage. Controlling for individual level variables diminished this effect for A1c, but not for acute or emergency care.

When considered in light of previous research showing associations between neighborhood factors and diabetes outcomes [5–10, 25], our findings suggest that comprehensive approaches to diabetes management need to include attention to neighborhood context [8, 38]. Failure to do so may help explain the continuing disproportionate diabetes burden in many neighborhoods despite decades of attention to individual-level clinical care and education. While randomized controlled trials changing neighborhood disadvantage are almost nonexistent [8], there are innovative ways to encourage social interaction in neighborhoods (increasing vegetation and common spaces [39], designing homes with porches or stoops [40]), and encourage self-care behaviors, such as physical activity, through improvements to infrastructure like lighting or sidewalks [41]. However, care must be taken to design such interventions in culturally appropriate and sensitive ways by engaging community members and securing buy-in from neighborhood residents [42].

In addition, we found that *both* neighborhood and individual-level factors contributed to outcomes of individuals with type 2 diabetes (rather than one or the other). Some of the effects of the individual-level variables on diabetes outcomes ran counter to our expectation, e.g., that greater numbers of comorbidities was associated with lower A1c values, that increasing diabetes empowerment was associated with higher A1c values, and that increasing age was associated with greater self-reported use of acute or emergency health care services. Regarding the first unexpected finding, it is possible that individuals with more comorbidities had more reason to seek care from their physician and thus received more care. It is also important to note that this association was weak (*p*-values ranging from 0.03 to 0.04). Regarding the later unexpected finding, it should be noted that some research suggests that younger adults with diabetes may have worse glycemic control than older adults, may be less likely to take medication prescribed for diabetes, and may be less likely to visit health care professionals for services like blood pressure and cholesterol checks [43, 44].

For the most part, these findings—of both individual and neighborhood level variables being important for health—suggest that targeting factors at individual and larger ecological levels will remain important. Failing to acknowledge human agency downplays the important role that individuals and practitioners may play in making important lifestyle and behavioral changes. At the same time, relying too heavily on only individual-level change neglects the powerful role that environments and context have in influencing individuals’ decisions and behaviors.

Multilevel level interventions, which target behavioral change at more than one ecological level [45], will remain important tools in improving health and reducing health disparities. Yet, most public health interventions are targeted at intrapersonal and interpersonal levels [46]. This is likely due to a number of reasons, including but not limited to: lack of training or resources for health professionals seeking to implement institutional, community, or policy-level programs; lack of theories or training in theories for creating interventions to change upper ecological levels; fewer metrics to evaluate changes at upper ecological levels; and added financial and logistical difficulty in trying to address upper ecological determinants. Transdisciplinary approaches, in which theories and methods are integrated across disciplines, may be particularly beneficial in disseminating lessons learned for future research on neighborhoods and health [47].

### Recommendations for future research

Based on the results of the present study, we identified three recommendations for future research. First, while we found that broad aspects of neighborhood disorganization encompassing economic, racial / ethnic diversity, and residential indicators were associated with A1c, which aligns with previous research finding NSD to be associated with metabolic syndrome among African American women [15], we found non-significant effects of neighborhood residential instability or ethnic heterogeneity on either A1c or self-reported use of acute or emergency health care services. It is possible that these constructs may not have been as important for our sample, which was composed of mostly older adults in the Southeastern U.S. Future research examining associations between NSD and diabetes outcomes in other settings and among other populations may be helpful, as well as critical investigation of ethnic heterogeneity as a construct.

Second, mediation analysis with these and other variables may be an important, underutilized tool for future research. Brown et al. has theorized that socioeconomic position (both at the neighborhood level and at the level of the individual relative to his or her position in the neighborhood) influences health through proximal mediators that include: health behaviors (e.g., diet/medication adherence, exercise), availability of and access to health care resources, and processes of care (i.e., technical and interpersonal care provided to patients within the health care setting) [48]. However, studies examining variables that mediate associations between neighborhoods and health are few and far between. For instance, in a systematic review examining associations between neighborhood characteristics and health outcomes among individuals with diabetes in the U.S., only 4 of the 38 identified studies conducted mediation analysis (Kowitt SD, Bhushan N, Fisher EB. Taking the “self” out of “self management”: a systematic review of the effects of neighborhood and community characteristics on diabetes outcomes in the United States, in preparation). Structural equation modeling and longitudinal studies will surely advance understanding of how neighborhoods affect health outcomes and which variables may act as mediators, confounders, or controls.

Finally, this is a novel study illustrating associations between neighborhood disadvantage and self-reported use of emergency or acute health care services among individuals with diabetes. This points to the importance of these factors in efforts to decrease avoidable emergency and acute or hospital care, a major priority of “bending the curve” through health care reform in the US as well as internationally. This study also builds upon other observations of the importance of neighborhood factors in avoidable care [49–51] In the present study, neighborhood disadvantage was evaluated at the level of the census tract. In contrast, some of the work of Brenner and colleagues in Camden New Jersey has explored hot spots defined at more micro levels, such as buildings and neighborhood blocks. Other researchers have proposed the idea of “spatial polygamy,” which refers to the idea that individuals are exposed to multiple contexts that interact to affect health (not just neighborhoods) [52]. Future research will address these various determinants and contexts, and importantly, will need to identify levels of influence that may be actionable at the level of individual or community interventions and policies.

### Limitations

We acknowledge several limitations. Most notably, our study design was cross-sectional, which limits our ability to infer causality. Second, while we included individual-level control variables (demographic, psychosocial, and clinical), we did not have a measure of individual-level income or insurance, which may have accounted for the observed effects especially of neighborhood economic disadvantage. While we included a measure of education, which has been used as a proxy of income in previous studies, further research controlling for income *and* examining interactions between neighborhood income and individual income will be important. Third, data came from a convenience sample of individuals in central North Carolina; findings may not generalize to other populations or settings. Fourth, level 1 residuals for one of our outcomes (A1c) appeared to be mostly normally distributed but there was evidence of a slight violation of normality, which could have biased results (e.g., biased fixed effects, standard errors, or variance components).

Fifth, our measure of use of acute and emergency health care services was self-reported and therefore subject to a number of potential biases, including information / recall bias. Some individuals may have incorrectly recalled how many times they had used a specific acute or emergency health care service. Additionally, the four items used to assess self-reported use of acute or emergency health care services were not mutually exclusive. A participant that reported “yes” to being seen in the ER may have also reported “yes” to having EMS being called and this would have been counted as two visits. Moreover, the questions used to ascertain acute or emergency care visits were not disease specific and could have been related to factors beyond diabetes. However, it is important to note that previous research has found self-reported hospitalization and emergency department visits to have high concordance with medical chart data and claims databases [53]. Supporting the validity of our measure, we also found that increasing number of comorbidities was associated with increased self-reported use of acute or emergency health care services. In addition, when we dichotomized the measure of use of acute / emergency health care services as 1 = any reported encounter or 0 = no reported encounter, both diabetes distress and high NSD were still associated with use (*p* < 0.05; data not shown).

Finally, while we cannot determine the temporality of observed associations (i.e., whether individuals who use emergency health care services were more likely to choose to live in more disadvantaged neighborhoods, or whether some aspect of neighborhood disadvantage caused people use more emergency health care services), our findings suggest that future research should explore this association. Our study is strengthened by our use of multilevel modeling techniques to control for any clustering within census-tracts and our inclusion of both demographic, psychosocial, and clinical variables in our models.

## Conclusions

Neighborhood and other ecological factors contributing to diabetes outcomes are poorly understood, yet growing research highlights the influence of neighborhoods and communities on management of diabetes as well as other chronic diseases. This research offers an in-depth exploration of how broad aspects of NSD are related to glycemic control and how economic disadvantage in particular is associated with avoidable use of acute and emergency health care.

## Abbreviations

A1c: Glycemic control
CI: Confidence interval
DES-SF: Diabetes Empowerment Scale-Short Form
ICC: Intraclass correlation
NSD: Neighborhood social disorganization
PAID: Problem Areas in Diabetes scale
SES: Socioeconomic status
